# Evaluation of the Effects of Platelet-Rich Plasma on Follicular and Endometrial Growth: A Literature Review

**DOI:** 10.5935/1518-0557.20210036

**Published:** 2021

**Authors:** Adrielli Riboldi Ferrari, Sylvia Cortrezzi, Edson Borges Junior, Daniela Braga, Maria do Carmo Borges de Souza, Roberto de Azevedo Antunes

**Affiliations:** 1 Fertility - Centro de Fertilização Assistida, São Paulo, SP, Brazil; 2 Fertipraxis, Human Reproduction Center. Rio de Janeiro, RJ, Brazil

**Keywords:** platelet-rich plasma, platelet-rich plasma, review

## Abstract

Platelet-rich plasma (PRP) has been used in several areas of medicine due to its ability to promote tissue regeneration by growth factors and cytokines. This review addresses the use of PRP to rejuvenate ovarian follicles and increase the thickness of the endometrium to receive an embryo. PRP is obtained from the patient's own blood (autologous blood) - a fact that determines a lower chance of rejection reactions. Alpha granules of platelets provide and release supra physiological amounts of growth factors and cytokines, which provide a regenerative stimulus in tissues with low healing potential. In the ovary, PRP and its growth factors stimulate vascularization and recruitment of available primordial follicles that could no longer be otherwise stimulated. The rejuvenation of the ovary by PRP infusion aims to obtain new oocytes in ovaries with low numbers of follicles or low follicular reserve markers. In the preparation of the endometrium, PRP is used for its several growth factors that allow tissue proliferation and endometrial thickening, especially in cases of endometria that are difficult to prepare or that fail to reach an adequate minimum thickness (>7mm). To date, there are few studies of greater expression in the literature that support the use of PRP with the two purposes described above. Thus, although promising, the technique must still be validated by larger clinical trials.

## INTRODUCTION

A recent increase in new autologous cycles of in vitro fertilization (IVF) and intracytoplasmic sperm injection (ICSI) has been reported by the latest report issued by the Latin American Society of Assisted Reproduction (REDLARA). It has been described that 40.9% of women undergoing new cycles were aged 35 to 39 years and that 31.1% were aged 40 years or older ^(Zegers-Hochschild *et al*., 2019)^. Infertility refers to the failure of conception by a couple and is known as a multifactorial syndrome in all cultures and societies ^(Alam *et al.,* 2019)^.

Infertility is common, with recent publications describing prevalence ranging between 9% and 18% in the general population ^(Aghajanova *et al.,* 2017)^. According to the National Summary Report of the Society for Assisted Reproductive Technology, a total of 43,098 IVF cycles were performed in 2018 (data: ^12^th^ SisEmbrio report, 2020^).

There is a growing body of data supporting the idea that infertility has implications that go beyond the patient's immediate reproductive needs. Approximately 5% to 10% of infertile women may have underlying genetic abnormalities, such as chromosomal aberrations, single or multiple genetic mutations and polymorphisms. A significant portion of infertility may be explained, at least in part, by exposure to environmental factors, endocrine disrupters and hormonal imbalances that not only may jeopardize reproductive health, but also increase later-life morbidity and mortality ^(Tarín *et al*., 2015)^.

The negative impact of infertility and its treatments is generally attributed to the stress caused by the inability to conceive, especially due to scheduled sexual intercourse, negative effects of the treatment (both on physical and psychological wellbeing), and pressure from family members and people around the couple. Compared to men, infertile women seem to be more stressed and more likely to develop sexual dysfunction, with great variability in their subjective experiences, as well as in the difficulties encountered during treatment ^(Facchin *et al*., 2019)^.

For ^Sills & Wood (2019)^, an intractable problem in clinical infertility is a low (or absent) ovarian reserve, which in turn reflects the natural depletion of oocytes associated with advancing maternal age, since the number of available oocytes has generally been considered finite and limited.

To increase follicular and tissue growth, platelet concentrates such as Platelet-Rich Plasma (PRP) have been used in tissue regeneration due to the high concentration of growth factors contained in platelets. When activated, platelets release high amounts of growth factors such as PDGF (platelet-derived growth factor), TGF-b1 (transformation growth factor b1), IGF-I (insulin-like growth factor-1) and VEGF (endothelial vascular growth factor), which promote cell proliferation and differentiation, chemotaxis and angiogenesis, thus optimizing the regenerative process ^(Lichtenfels, 2012)^.

The application of PRP in a reproductive context was an innovation considered for the first time only a few years ago by Pantos *et al*. (2019), when a group of patients with poor prognosis infertility received intra-ovarian injections of PRP followed by in vitro fertilization with their own oocytes ^(Sills & Wood, 2019)^.

Knowing that adequate endometrial thickness is a critical factor for embryo implantation, ^Chang *et al*. (2015)^ conducted a study that investigated the effects of PRP in women with thin endometria (<7mm). In its encouraging results, a significant increase in endometrial thickness was shown in individuals given PRP infusion compared to controls (not administered PRP).

Treatment with PRP might improve the outcomes of patients with decreased endometrial thickness (<7mm) undergoing IVF cycles and even of individuals on hormonal therapy. One of the most important phases of Assisted Reproductive Technology (ART) treatment is, undoubtedly, embryo transfer and implantation; successful therapy requires, in addition to a competent embryo, a receptive endometrium. The objective of this paper is to review literature on the effects of PRP on follicular and endometrial growth.

## MATERIALS AND METHODS

The articles featured in this literature review were searched on PubMed and Google Scholar database and included papers published in the period of 2009-2019 in Portuguese and English. The following keywords in English were used: "PRP AND human fertilization", "PRP AND growth factor", "PRP AND follicle growth", "PRP AND ovarian", "PRP AND folliculogenesis", "Platelet-rich plasma", "Platelet-rich plasma AND endometrium"; and the following keywords in Portuguese were used: "crescimento folicular e endometrial", "plasma rico em plaquetas na reprodução humana", "plasma rico em plaquetas". Additional papers were searched from the references cited in the found articles (Table 1).

**Table 1:** Brief description of the main authors and their characteristics for the construction of the bibliographic review.

AUTHORS/YEAR	OBJECTIVE	DATABASE	KEYWORDS	INCLUSION CRITERIA
Farimani *et al*. (2019)	Intra-ovarian injection in women with POR.	PubMed	“PRP AND follicle growth”, “PRP AND ovarian”, PRP AND folliculogenesis”, “Platelet-rich plasma”.	It matches the sought subject; text in English; time period; included humans.
Pantos *et al*. (2019)	Case report of women with ovarian failure.	PubMed, Google Scholar	“PRP AND follicle growth”, “PRP AND ovarian”, PRP AND folliculogenesis”, “Platelet-rich plasma”.	Text in English; case report recently published including humans.
Kim *et al*. (2019)	Use of PRP in thin endometrium	PubMed	“autologous PRP AND endometrium”	Prospective study describing the PRP preparation technique.
Sfakianoudis *et al*. (2019)	PRP infusion and ovarian response	PubMed	“PRP AND follicle growth”, “PRP AND ovarian”, PRP AND folliculogenesis.”	Publication within the chosen time period; text in English; study with humans.
Molina *et al*. (2018)	Endometrial preparation with PRP	PubMed	“PRP AND endometrial preparation”	Brazilian publication; optimistic results; study enrolled women.
Jang *et al*. (2017)	Injection of intrauterine PRP in 60 rats.	PubMed	“PRP AND endometrium”	Publication of experimental character and of good relevance.
Zadehmodarres *et al*. (2017)	Intrauterine PRP injection in 10 women	PubMed	“PRP AND endometrium”	Pilot study, but with several citations in relevant studies.
Farimani *et al*. (2016)	Intrauterine administration of PRP.	PubMed, Google Scholar	“PRP AND ovarian”, “crescimento folicular e endometrial”	Publication translated from English into Portuguese, with reports of studies in humans.
Chang *et al*. (2015)	Report of minimum endometrial thickness and intrauterine administration of PRP.	PubMed, Google Scholar	“PRP AND ovarian”, “Platelet-rich plasma AND endometrium”, “crescimento folicular e endometrial”	Renowned publication; text in English; report of studies in humans.

After searching the databases for articles and applying the defined search strategies, the articles were read and translated from the original version only after determining whether, based on their abstracts, the studies met the eligibility criteria; only then were they included in the review.

## LITERATURE REVIEW

### Folliculogenesis

Ovarian follicles are structures generated in the ovaries that have two major functions, the production of hormones and gametes. Ovarian follicles may develop until ovulation or undergo atresia though the control of the hypothalamic-pituitary-gonadal axis ^(Zhou *et al*., 2019)^. The reason why some oocytes develop and others in the same cohort undergo atresia is unknown ^(Britt, 2008)^.

However, it is known that early antral follicles do not necessarily grow coordinately in response to exogenous gonadotropins to achieve simultaneous functional and morphological maturation, and that not necessarily all follicles that respond to FSH express sufficient LH receptors to respond to the signal from maturation ^(Kahraman *et al*., 2017)^.

Due to hypersecretion of LH causing premature oocyte maturation, the decline in female infertility has been mitigated, as younger oocytes have a better balance of cellular activity compared to aged oocytes. During the process of folliculogenesis, the oocyte undergoes a significant set of genetic, epigenetic and cytoplasmic changes in order to attain proficiency in fertilization. This entire course of events depends on a continuous interaction between oocytes and granulosa cells that safeguard the coordination of all events sequenced in the ovary under the influence of paracrine and endocrine factors ^(Alam *et al*., 2019)^.

### Growth Factors Found in PRP (Figure 1)

**Figure 1:**
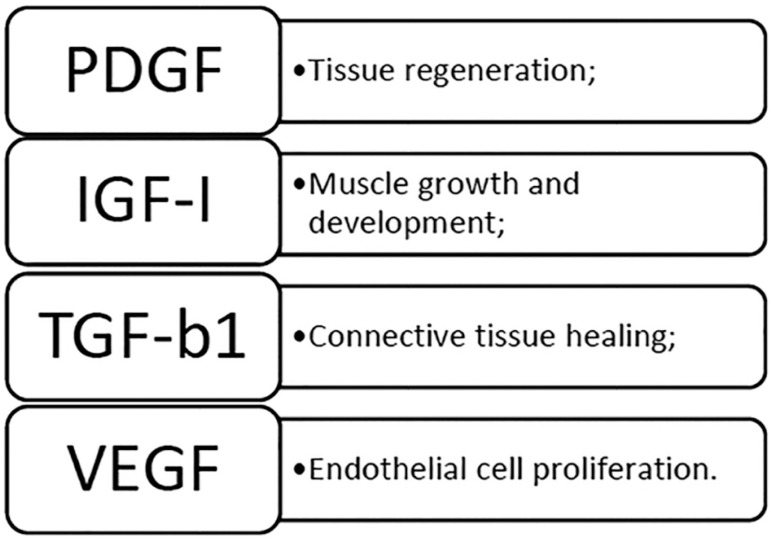
Most common growth factors in PRP and their main functions.

Platelets are known for their main function of preventing blood loss in vascular injury sites through interactions between platelets and proteins, which cause the formation of fibrin clots. ^(Fabi & Sundaram, 2014)^. The chemical composition and morphology of platelets is understood as presenting an outer portion, the phospholipid membrane, and an inner portion where microtubules, dense granules, α-granules, dense tubular system, mitochondria, glycogen reserve are found. Platelets interfere with primary hemostasis through the properties of their membranes and the content of their cytoplasmic organelles ^(Grassi & Araujo, 2012)^.

PDGF is very important because it is the first component to be present at the injury site; it is also one of the most abundant factors present in PRP preparations together with TGF, which aid in the migration and proliferation of human osteoblast cell lines ^(Casati *et al*., 2014)^. PDGF is known to increase cell recruitment, proliferation and differentiation, although it also plays a role in angiogenesis and inflammation ^(Du *et al*., 2018)^. This factor has been used to accelerate tissue regeneration, alone or in combination with other growth factors.

IGF-I, also known as sulfation factor and somatomedin C, is composed of 70 amino acids and is a member of the insulin family. Its function is to stimulate production, differentiation, migration, survival and metabolism. It acts through IGF-1R, which is predominant in the body, where it belongs to the family of tyrosine kinase receptors and is phosphorylated by IGF-I ^(Deochand *et al*., 2016)^.

One of the potential chemo attractants includes transforming growth factor β1 (TGF-β1). TGF-b1 may be involved in signaling events related to proliferation, differentiation and recruitment of parents to the injury site, in order to initiate regeneration, acting as a stimulating factor for tissue repair ^(Gonçalves *et al*., 2016)^.

TGF-β1 stimulates VEGF expression and secretion of immortalized human granulosa-lutein (hGL) cells and primary hGL cells. The levels of TGF-β1 and VEGF protein are increased in the follicular fluid of patients, and the levels of these two proteins in the follicular fluid have been positively correlated ^(Fang *et al*., 2020)^.

VEGF, a secreted protein specific to the endothelium, plays an important role in angiogenesis ^(Zhang *et al*., 2016)^. A study by ^Nayak & Dinda (2015)^ observed a peak of VEGF at the beginning of the proliferative phase and in the glands during the secretory phase favoring the pre-implantation period due to the action of progesterone.

### Platelet-Rich Plasma (PRP)

PRP is a plasma preparation enriched with a platelet concentration above that normally contained in whole blood ^(Malanga & Goldin, 2014)^. Protocols include one or two centrifugation steps to separate whole blood into three layers: an upper layer of plasma, a buffy coat, and red blood cells (RBC) at the bottom. The rationale for the use and therapeutic potential of preparations with a high concentration of platelets is based on their ability to supply and release supra physiological amounts of essential growth factors and cytokines from their alpha granules to provide a regenerative stimulus that increases healing and promotes repair in tissues with low healing potential ^(Davis *et al*., 2014)^.

The main functions of platelets include the prevention of acute blood loss and the repair of vascular walls and surrounding tissues after injury. During healing, platelets are activated and aggregated to release granules containing growth factors that stimulate the inflammatory cascade and the healing process. Through the activation of platelets in PRP, cytokines and growth factors become bioactive and are secreted within ten minutes of coagulation. They can regulate cell migration, fixation, proliferation and differentiation and promote the accumulation of extracellular matrix ^(Chang *et al*., 2015)^.

During the initial stages of wound repair, activated platelets attract and stimulate cell migration to the wound, aggregating and forming a fibrin matrix. This matrix then serves as a tissue scaffold for the sustained release of platelets and cytokine growth factors, which stimulate cell recruitment, differentiation and communication ^(Scherer *et al*., 2012)^.

^Borzini & Mazzucco (2007)^ reported in their literature review article that the application of PRP varies in various clinical settings involving skin, bone, dental, maxillofacial surgery, diabetic foot and leg, cardiac and vascular surgery, tympanic, eye and corneal lesions, nerve injuries, spinal fusion, burns, cosmetic surgery and facelifts. Most of these studies have shown positive, encouraging results. Despite the evidence on the biological mechanisms that support the clinical efficacy of platelets, it should be considered that the technical aspect of PRP preparation is closely related to its clinical effects. Therefore, lack of standardization of PRP preparation techniques in different studies may be related to the lack of effectiveness described by some authors.

The wide variability in PRP formulations creates a challenge to accurately extract conclusions from the literature to guide PRP production and determine indications for use, leading to the development of PRP classification schemes to facilitate investigations in clinics ^(Delong *et al*., 2012; Mishra *et al*., 2012)^.

Recently, ^Dawood & Salem (2018)^ presented the steps of a representative method of preparing the PRP. However, PRP preparations can be classified according to the preparation method, the sample content and the proposed application. Preparations vary in terms of centrifugation speed, centrifugation time and use of anticoagulants, while the content varies according to the predominant constituent (e.g.: platelets, leukocytes or growth factors).

The methodologies for PRP preparation are still under development, and different preparation protocols have emerged, from conventional blood centrifugation to commercial systems such as activation by the addition of collagen, calcium and/or thrombin, by contact with the glass tube, or by cycles of cryopreservation ^(Mazzucco *et al*., 2009)^.

^Roh *et al*. (2016)^ demonstrated that PRP activated with a mixture of thrombin and calcium significantly increased the release of growth factors over seven days compared with non-activated PRP. However, there is uncertainty regarding the speed ^(Scherer *et al*., 2012)^.

Activation causes the granules present in the platelets to fuse with their cell membrane (also called degranulation), where secretory proteins (for example, PDGF, TGF-β etc.) are transformed into a bioactive state by the addition of histones and side chains of carbohydrates. The active proteins are then secreted, binding to the transmembrane receptors of the target cells, which include mesenchymal stem cells, osteoblasts, fibroblasts, endothelial cells and epidermal cells. The agonists bind to transmembrane receptors, then activate an intracellular signal protein that causes the expression of a sequence of genes that directs cell proliferation, matrix formation, osteoid production, collagen synthesis, etc., thus causing repair and tissue regeneration ^(Dhurat & Sukesh, 2014)^.

No evidence has been found about the ideal activator concentration needed to trigger the optimal release of growth factors during the PRP activation process, and different concentrations may therefore lead to different results ^(Jo *et al*., 2013)^.

Despite these variations, all protocols follow a generic sequence consisting of blood collection, initial centrifugation to separate red blood cells, subsequent centrifugation cycles to concentrate platelets and other components, and the activation of the sample by adding a platelet agonist ^(Dhurat & Sukesh, 2014)^.

### Effect of PRP on Follicular Growth

PRP therapy has been investigated in women with premature ovarian failure (POF), infertile women over 35 and women with a low ovarian reserve. In this procedure, PRP is injected into the ovary under ultrasound guidance ^(Dawood & Salem, 2018)^.

Until 2011, there was no clear definition of poor responder patients (PORs), which led to a significant degree of confusion. However, the introduction of the Bologna criteria allowed the description of a very heterogeneous group of patients with very different success rates after ART. This has led to the recent development of the POSEIDON (Patient-Oriented Strategies Encompassing Individualized Oocyte Number) criteria for POR, which stratified patients into more homogeneous subgroups and, more importantly, produced recommendations for clinical management. The treatment of patients with POR requires an individualized approach ^(Haahr *et al*., 2018)^.

The Bologna criteria published in 2011 by the European Society for Human Reproduction and Embryology (ESHRE) were established mainly to define the population with POR based on strict criteria. The underlying idea was that this would allow the definition of more homogeneous groups when new treatments for POR were compared ^(Ferraretti *et al*., 2011)^. Although the Bologna criteria were a crucial step in defining POR, it was clear that the criteria did not adequately take into account the age-related impacts on oocyte quality, which obviously affects success rates ^(Haahr *et al*., 2018)^. The POSEIDON classification subdivides patients into four subgroups based on quantitative and qualitative parameters ^(Haahr *et al.,* 2018)^.

^Sfakianoudis *et al*. (2019)^ described ovarian PRP infusion as an efficient model to determine the successful management of patients with poor ovarian response. Given the highly angiogenic structure of the ovary and the critical role of various platelet-derived factors in vascular activation and stabilization, treatment with PRP might likely enrich the dysfunctional ovarian tissue of patients with factors essential for neoangiogenesis, leading to tissue regeneration and reactivation.

In an investigation about intra-ovarian PRP injection in women with POR, a study by ^Farimani *et al*. (2019)^ reported an increase in the average number of oocytes before and after PRP injection from 0.64 to 2.1. The results of this study appear to be the first to report on the effects of intra-ovarian PRP injection on the ovarian response to controlled ovarian stimulation.

In a recently published report, ^Pantos *et al*. (2019)^ analyzed patients with a history of implantation failure after IVF who rejected the option of having donor eggs and received treatment with autologous ovarian PRP; pregnancies by natural conception were achieved within two to six months of treatment with PRP.

Studying rats with polycystic ovary syndrome, ^Seyyed Anvari *et al*. (2019)^ demonstrated that PRP infusion decreased follicular atresia and notably improved follicular growth.

Considering the angiogenic composition of the ovary and the fundamental influence of PDGF on vascular activation and stabilization, treatment with autologous PRP may be seen as a facilitator of regeneration of ovarian tissue ^(Bos-Mikich *et al*., 2018)^.

### Effect of PRP on Endometrial Thickness

Successful embryo implantation requires a good quality embryo and a receptive endometrium. It was found that adequate endometrial growth is necessary for successful implantation ^(Colombo *et al*., 2017)^.

The endometrium plays a pivotal role in implantation and pregnancy. The pregnancy rate increases as endometrial thickness grows. For ^Chang *et al*. (2015)^, the minimum endometrial thickness necessary for embryo transfer is 7mm at the end of the follicular phase. ^Noyes e*t al*. (1995)^ reported a significantly higher pregnancy rate (48.6%) in patients with an endometrial thickness >9mm, compared with 16% in individuals with an endometrial thickness <9mm (apud ^Coksuer *et al*., 2019^).

Intrauterine PRP infusion is a new approach that has been suggested for the treatment of recurrent implantation failures ^(Chang *et al*., 2015)^. Although it is not clear how intrauterine PRP administration acts to increase endometrial thickness, studies have found a positive increase in endometrial thickness, leading to higher pregnancy rates.

A pilot study by ^Zadehmodarres *et al*. (2017)^ included 10 patients with a history of cycle cancelation due to decreased endometrial thickness (<7mm). Endometrial thickness increased 48 hours after the first PRP application and reached more than 7mm after the second PRP application in all patients. Embryo transfers were performed in all patients; five became pregnant and four pregnancies progressed normally. PRP enabled endometrial growth in patients with thin endometria.

^Chang *et al*. (2015)^ administered intrauterine PRP to infertile women with thin endometria and reported that endometrial thickness increased >7mm within 48-72 hours of PRP infusion in all five patients. All patients became pregnant, with four pregnancies progressing to full term. In a pilot study, ^Farimani *et al*. (2016)^ performed intrauterine administration of PRP 36 hours prior to frozen embryo transfers. The six women included in the study became pregnant.

Despite the lack of specific studies evaluating the effects of PRP on the endometrium, ^Jang *et al*. (2017)^ analyzed damaged endometrial tissue of 60 rats and found faster endometrial regeneration after PRP injection. This shows that its main growth factor, PDGF, plays an important role in cell proliferation within the endometrium.

^Kim *et al*. (2019)^ reported the use of PRP in women with refractory thin endometrium. The authors clearly described the techniques used to prepare PRP, including the amounts of sample used and the speed of centrifugation. This study, which included women with two or more IVF attempts and refractory thin endometria, reported improved implantation, pregnancy and live birth rates. Treatment with intrauterine PRP seems not only to promote endometrial growth in patients with thin endometria, but also to positively affect assisted reproduction outcomes.

## CONCLUSION

PRP has positive, promising impacts on endometrial and follicular growth with the added benefit of using autologous blood, which decreases the chances of adverse reactions and effects. PRP has become an alternative for older women with low follicular reserve and unresponsive endometria who wish to become mothers. The outcomes must be further evaluated to confirm the safety and effectiveness of PRP in gynecology.
